# Late-onset or chronic overweight/obesity predicts low self-esteem in early adolescence: a longitudinal cohort study

**DOI:** 10.1186/s12889-021-12381-5

**Published:** 2022-01-06

**Authors:** Wei Jie Gong, Daniel Yee Tak Fong, Man Ping Wang, Tai Hing Lam, Thomas Wai Hung Chung, Sai Yin Ho

**Affiliations:** 1 School of Nursing, Li Ka Shing Faculty of Medicine, University of Hong Kong, 4/F, 21 Sassoon Road, Pokfulam, Hong Kong, China; 2 School of Public Health, Li Ka Shing Faculty of Medicine, University of Hong Kong, 7 Sassoon Road, Pokfulam, Hong Kong, China; 3 Student Health Service, Department of Health, 4/F, Lam Tin Polyclinic, 99 Kai Tin Road, Kwun Tong, Kowloon, Hong Kong, China

**Keywords:** Developmental trajectory, Overweight, Obesity, Self-esteem, Adolescent, Longitudinal study

## Abstract

**Background:**

How weight status changes with time may affect self-esteem was seldom studied. We identified the distinct trajectories of overweight/obesity over age and assessed their associations with different domains of self-esteem in Hong Kong Chinese children.

**Methods:**

Territory-wide longitudinal data of 48,558 children (girls: 50.0%; 6.3 ± 0.51 years) studying Primary 1 in the academic cohorts of 1995/96 and 1996/97 followed till Primary 6 were obtained from the Student Health Service of Hong Kong. Weight was annually measured and categorized as underweight/normal and overweight/obese and self-esteem was measured in Primary 6. Distinct trajectories of weight status were first identified using growth mixture modeling and their associations with low self-esteem were assessed by logistic regression.

**Results:**

Four distinct overweight/obesity trajectories were identified: never (76.8%), late-onset (8.1%), early-onset (4.2%) and chronic (10.9%) overweight/obesity. Compared with children who were never overweight/obese, more of those in the late-onset or chronic overweight/obesity group showed low self-esteem and specific domains including general, social and academic/school-related (adjusted odds ratios: 1.20 - 1.43, all *P* < 0.001) except parent/home-related self-esteem (*P* = 0.36), whereas children being in the early-onset overweight/obesity group showed no significant difference (*P* ≥ 0.53) except a lower risk of low social self-esteem (adjusted odds ratio = 0.82, *P* = 0.03).

**Conclusions:**

Late-onset or chronic overweight/obesity predicted low general, social and academic/school-related self-esteem. Children who successfully reduced weight may have equal levels of self-esteem or even better social self-esteem than those being always underweight/normal weight. Overweight/obese children had a vulnerability to self-esteem in non-domestic environments.

**Supplementary Information:**

The online version contains supplementary material available at 10.1186/s12889-021-12381-5.

## Background

Self-esteem refers to a person’s evaluation of or attitude towards him/herself [1] and is a fundamental aspect of mental health [2]. Low self-esteem is associated with negative psychological outcomes in young people, such as depression, anxiety, loneliness [3, 4], as well as problematic behaviors, including suicide attempts, substance abuse, and risky sexual activity in childhood [5, 6]. The adverse outcomes could even persist in adulthood, leading to poor health, engagement in criminal activities and confined economic conditions [7]. To promote healthy child development, special attention to children’s self-esteem is necessary.

Childhood obesity has become a severe global public health threat, with 213 million overweight and 124 million obese children worldwide in 2016, corresponding to a tenfold increase since 1975 [8]. Children with excess weight are more likely to be stigmatized and have psychosocial problems. However, the association between childhood obesity and self-esteem were found to be inconsistent in both cross-sectional [9, 10] and cohort studies [11, 12], including no association between the change of body mass index (BMI) z-score with self-esteem in 6,520 Hong Kong adolescents [11], and a negative association between high BMI growth with lower scores of self-esteem in 16,936 UK adolescents [12]. Possible reasons include the dynamic changes in weight status [13] and differences in sample size or ethnicity, indicating the need for large-scale population-based longitudinal studies on different ethnic groups. Nevertheless, while assessing BMI would be useful to depict the level of changes over time, it may not reflect the change of weight status. A child with increasing BMI may not necessarily have BMI reaching overweight or obesity. BMI and BMI z-score are often modelled using the mean values, which is vulnerable to the influence of extreme values. Their trajectories were found to be substantial consistency across time [14]. Hence, weight status should be a better alternative for assessing the impact of weight changes on self-esteem.

Self-esteem is a multidimensional construct corresponding to different aspects of children’s daily life. Children may have different levels of self-esteem domains related to their physical appearance, peer relationships and academic achievement [5, 15]. Understanding of different self-esteem domains can guide practical efforts to optimize children’s psychosocial development. Middle childhood, age 6-12 years old [16], is a crucial period for the development of self-esteem, during which children undergo critical physical, cognitive and social changes [17]. To our knowledge, no studies have reported the multi-dimensionality of self-esteem when assessing the association with weight change at middle childhood. Therefore, this study aimed to identify the distinct trajectories of weight status over age and assessed their associations with different domains of self-esteem in Hong Kong Chinese children.

## Methods

### Study design

This population-based retrospective cohort study included students studying Primary 1 (P1, equivalent to US Grade 1) during the academic years of 1995/96 and 1996/97 and were followed annually till their Primary 6 (P6). Anonymous data were obtained from the Student Health Service (SHS), Department of Health, Government of Hong Kong SAR. The SHS provided a free voluntary annual health assessment program on physical and psychological health for primary school students since 1995/96 and for secondary school students since 1996/97, with an enrollment rate of 78.6% (provided by SHS). Enrolled students in all grades were invited to receive an annual health assessment and to complete a biennial standardized self-administered questionnaire on lifestyles and psychosocial health from Primary 4. All data were stored in the SHS database, with each participant having a unique identification number. Details of the service have been reported elsewhere [18]. The study protocol was approved by the Institutional Review Board of The University of Hong Kong/Hospital Authority Hong Kong West Cluster and The Department of Health Ethics Committees.

### Measurements

Children’s weight (to the nearest 0.1 kg) and height (to the nearest 0.1 cm) were annually measured by well-trained healthcare workers or nurses according to a standard protocol. BMI was calculated as weight divided by squared height (kg/m^2^), and weight status was defined by using age and sex-specific BMI references according to the International Obesity Task Force (IOTF) Standards [19]. Children’s socioeconomic status was indicated by the parental educational level and occupation.

This study used self-esteem assessed at P6 by the 60-item Culture-Free Self-Esteem Inventories-Second Edition (CFSEI-2), which has been shown to be a reliable and valid tool to assess the self-esteem of children in Hong Kong [20, 21]. It comprised a total score as well as four subscale scores that covered four domains of self-esteem: 1) general self-esteem, reflecting children’s overall perception of their own worth; 2) social self-esteem, reflecting children’s perception of their quality of peer relationship, 3) academic/school-related self-esteem, reflecting children’s perception of their ability to achieve academic success, and 4) parent/home-related self-esteem, reflecting children’s perception of how their parents/ caregivers view them and their status at home [18, 20]. The Cronbach’s α for the CFSEI-2 total score was 0.80 in this study. For the general self-esteem domain, subscale scores ≤ 7 or ≤ 10 were considered as “very-low” or “low” respectively. For the other domains, scores ≤ 2 or ≤ 4 were considered as “very-low” or “low” respectively. Overall, children were considered to have low self-esteem if they had a total score ≤ 19 or a “very-low” score in any domain [20]. In addition, a lie score comprising the last 10 items of the CFSEI-2 questionnaire were also calculated for indicating the defensiveness in children’s self-reported answers. Records with a lie score ≤ 2 were regarded as unreliable [20].

## Statistical analysis

Two academic cohorts of students were identified from P1 in 1995/96 and 1996/97 till P6 in 2000/01 and 2001/02 respectively. To assess the association of weight status changes with self-esteem, only participants with at least two measurements of weight and height from P1 to P6 and one measurement of self-esteem in P6 were included. Unreliable records with a lie score ≤ 2 were removed from the analysis [20]. The self-esteem scores of the included students were compared with that of the corresponding normative sample from Hong Kong [21] using one-sample t-tests.

We used dichotomized weight status as underweight/normal and overweight/obesity to identify the distinct trajectories that well covers patterns of over-age weight status changes from age 6 to 11 years, using the semiparametric growth mixture modeling by the SAS Proc Traj procedure [22]. Sex, cohort and parental educational level and occupation were adjusted to avoid their potential influences on the trajectories [23]. Quadratic curves were estimated by a series of logit models, with a sequential increase of the number of trajectory groups from two to six. The number of trajectory groups was taken as the one that minimized the Akaike information criteria (AIC), Bayesian information criterion (BIC), and sample-size adjusted BIC (ssBIC) and maximized entropy (a measure of how well all cases are classified) of the model, while using the Lo-Mendell-Rubin likelihood ratio (LMR) tests to compare whether k to k-1 classes provide better model fit [24]. Each child was then assigned to the trajectory group that best fits his/her weight status change pattern by the highest estimated group-membership probability. Age-specific percentages of overweight/obesity were calculated for different trajectory groups. The association of children’s characteristics with the identified trajectory groups was assessed using multinomial logistic regression. The goodness of fit was assessed by the generalized Hosmer-Lemeshow tests using the generalhoslem.package in R 3.5.3 [25].

The influences of weight status change on self-esteem in P6 were examined by logistic regression with trajectory group as the covariate, after adjusting for sex, cohort and parental educational level and occupation. Adjusted odds ratios (AORs) with 95% CIs were reported using binomial logistic regression. The Hosmer-Lemeshow test was applied to assess each models’ goodness of fit [26]. All logistic models were estimated using the SAS Proc Logistic procedure.

Considering the fact that underweight children, especially boys, were reported to have a higher risk of low self-esteem than their normal-weighted peers [27, 28], sensitivity analysis was conducted after excluding children who were underweight in P6. Other than using the mean age for each time point, we also repeated the sensitivity analysis only using children who were 6-year-old in P1 and 11-year-old in P6. Except for the generalized Hosmer-Lemeshow test, all the other analyses were conducted using the Statistical Analysis System (SAS Institute, Cary, NC, US) or R 3.5.3, and a two-tailed significance level of 0.05 was used.

## Results

### Sample characteristics

Totally 50,005 students were extracted from the original database who were studying P1 in 1995/96 and 1996/97 and tracked till P6 in 2000/01 and 2001/02 respectively. After excluding those without at least 2 BMI measurements from P1 to P6 or self-esteem data in P6 or those with a lie score ≤ 2, 48,558 (97.1%) students at age of 6.3 ± 0.51 years in P1 (girls: 50.0%) were included in the analysis, and 92.6% of them had 5 or more measurements of BMI (Supplementary Table 1). The included children had the same level of self-esteem with the corresponding normative sample in Hong Kong (all *P* ≥ 0.05), except for the social subscale (Table 1).

**Table 1:** Comparisons of self-esteem between the study sample and the corresponding normative sample in Hong Kong (Mean±SD)

Self-esteem	Female			Male		
	Study sample (*N* = 24,297)	The normative sample	t-value, *P*^a^	Study sample (*N* = 24,261)	The normative sample	t-value, *P*^a^
Total	38.03 ± 6.74	36.16 ± 6.67	1.94, 0.052	37.12 ± 7.16	35.42 ± 6.38	1.64, 0.100
General	15.38 ± 3.15	14.86 ± 2.98	1.15, 0.248	15.10 ± 3.24	14.25 ± 2.98	1.82, 0.069
Social	6.99 ± 1.76	6.35 ± 1.65	2.54, 0.011	6.90 ± 1.81	6.38 ± 1.12	3.21, 0.002
Academic/school-related	6.99 ± 1.77	6.69 ± 1.75	1.19, 0.236	6.86 ± 1.86	6.67 ± 1.77	0.71, 0.480
Parent/home-related	8.67 ± 1.87	8.27 ± 1.69	1.50, 0.135	8.26 ± 2.17	8.13 ± 2.24	0.41, 0.678

^a.^*P* was calculated using one-sample t-tests.

### Weight status trajectories

**Fig. 1:**
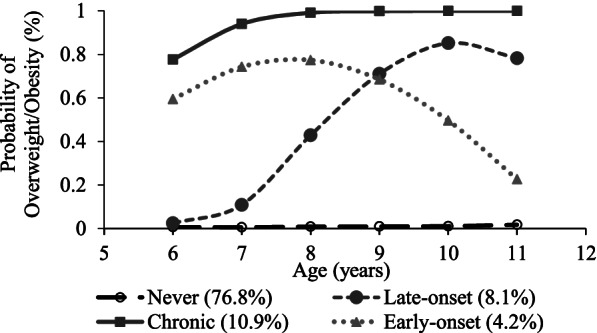
Developmental trajectories of overweight/obesity in Hong Kong children (*N* = 48,558)

The four-group model was the best-fitted trajectory model that simultaneously minimized the AIC, BIC, and ssBIC at -65,729.04, -65,705.58, and -65,586.91, and maximized entropy at 0.935, respectively, with improved model fit from the three-group model (LMR test: *P *< 0.001) and the improvement from the five-group model being statistically insignificant (LMR test: *P *= 0.19) (Supplementary Table 2). The four distinct overweight/obese trajectories were identified which best characterized the complex developmental course (Figure 1), and they were labelled as ‘never’ (76.8%), ‘late-onset’ (8.1%), ‘chronic’ (10.9%), and ‘early-onset’ (4.2%). The never-overweight/obesity group consisted of children who were never or rarely overweight/obese during the study ages. The late-onset overweight/obesity group included children whose probability of overweight/obesity began to raise at approximately 7 to 8 years. The early-onset overweight/obesity group included children whose probability of overweight/obesity dropped down at approximated 9 to 11 years. The chronic overweight/obesity group consisted of children who had a high probability of overweight/obesity throughout the study ages.

Children in late-onset and chronic overweight/obesity groups were mostly boys (67.8% and 61.3% respectively) and had lower-educated parents (91.6% and 91.7% respectively), and had higher rates of low total self-esteem, general, social, academic/school-related self-esteem (4.3 - 11.3%) than those in the never and early-onset overweight/obesity groups (2.8 - 9.7%) (all *P* ≤ 0.003) (Table 2).

**Table 2:** Children’s characteristics by overweight/obesity trajectories (*N* = 48,558)

Characteristics	Never (*n* = 37,578)	Late-onset (*n* = 3862)	Chronic (*n* = 5380)	Early-onset (*n* = 1738)	*P*^a^
Age, Mean±SD (years)	6.3 ± 0.51	6.3 ± 0.53	6.3 ± 0.51	6.3 ± 0.50	0.55
Sex, %					< 0.001
Female	53.2	32.2	38.8	55.9	
Male	46.8	67.8	61.2	44.1	
Parental educational level, %					0.003
Tertiary	9.2	8.4	8.3	10.8	
Secondary	73.4	74.1	74.4	73.4	
Primary/below	17.4	17.5	17.3	15.8	
Parental occupation, %					0.07
Managerial/professional	20.9	21.0	21.0	21.9	
Clerical/service industry	31.3	31.7	32.0	31.1	
Manual job	45.3	44.3	45.0	44.9	
Unemployed	2.5	3.0	2.0	2.1	
Cohort, %					< 0.001
1995/96	50.5	51.0	48.9	46.0	
1996/97	49.5	49.0	51.1	54.0	
Low self-esteem rate in P6, %					
Total	3.1	4.7	4.3	2.8	< 0.001
General	8.7	12.3	12.3	7.9	< 0.001
Social	9.3	11.3	12.0	7.7	< 0.001
Academic/school-related	9.7	12.1	12.3	9.1	< 0.001
Parent/home-related	6.2	7.6	7.1	6.2	< 0.001

^a^Based on Analysis of variance (ANOVA) tests for age, and Chi-square tests for other variables.

Table 3 shows that compared with girls, boys had a significantly higher odds of late-onset (AOR = 2.40, 95% CI 2.24 - 2.58, *P* < 0.001) and chronic (AOR = 1.80, 95% CI 1.69 - 1.91, *P* < 0.001) overweight/obesity, and a lower odds of early-onset overweight/obesity (AOR = 0.90, 95% CI 0.81 - 0.99, *P* < 0.03). Compared with children having tertiary-educated parents, those having lower-educated parents had a higher odds of being in the late-onset and chronic overweight/obesity group (AORs: 1.17 - 1.19; all *P* ≤ 0.03), but not in the early-onset group (AORs: 0.72-0.82; all *P* ≤ 0.03). For parental occupation, children with unemployed parents had a lower odds of being chronically overweight/obese than those having parents working in managerial/professional levels (AOR = 0.75, 95% CI 0.60 - 0.93, *P* = 0.009), but no statistically significant difference was found in other trajectories (*P* ≥ 0.19). The generalized Hosmer-Lemeshow test showed *P* = 0.89, which indicated the model was adequate.

**Table 3:** Associations of Primary 1 characteristics with overweight/obesity trajectories (*N* = 48,558)

Characteristics	Late-onset	Chronic	Early-onset
Sex			
Female	Reference	Reference	Reference
Male	2.40 (2.24, 2.58)***	1.80 (1.69, 1.91)***	0.90 (0.81, 0.99)***
Parental educational level			
Tertiary	Reference	Reference	Reference
Secondary	1.17 (1.03, 1.35)*	1.17 (1.04, 1.32)**	0.82 (0.69, 0.98)*
Primary/below	1.19 (1.02, 1.40)*	1.18 (1.02, 1.35)**	0.72 (0.58, 0.90)**
Parental occupation			
Managerial/professional	Reference	Reference	Reference
Clerical/service industry	0.98 (0.88, 1.09)	0.98 (0.90, 1.07)	1.00 (0.87, 1.16)
Manual job	0.94 (0.85, 1.04)	0.94 (0.87, 1.03)	1.03 (0.89, 1.19)
Unemployed	1.17 (0.94, 1.45)	0.75 (0.60, 0.93)**	0.78 (0.54, 1.14)
Cohort			
1995/96	Reference	Reference	Reference
1996/97	0.98 (0.91, 1.04)	1.06 (1.00, 1.13)*	1.20 (1.09, 1.32)*

Results are presented as adjusted odds ratios (95% confidence interval) from multinomial logistic regression, with the never overweight/obesity trajectory taken as the reference category. *P < 0.05; **P < 0.01; ***P < 0.001.

### Influences of weight status trajectories on self-esteem in P6

Table 4 shows that compared with never-overweight/obese children, those who were in the late-onset or chronic overweight/obesity group had higher odds of low self-esteem and three specific domains, including general self-esteem, social self-esteem and academic/school-related self-esteem (AORs: 1.20 - 1.43, all *P* < 0.001). In contrast, children who were in the early-onset overweight/obesity group showed no significant difference in self-esteem (*P* = 0.53) except a lower odds of low social self-esteem (AOR = 0.82, 95% CI 0.68 - 0.98, *P* = 0.03). Compared with chronically overweight/obese children, those who were in the late-onset overweight/obesity group showed no statistically significant difference in the odds of being low self-esteem as well as its four domains (*P* ≥ 0.21). In addition, parent/home-related self-esteem was the only domain that was found to show no association with different trajectories (*P* = 0.36). The Hosmer-Lemeshow test showed all *P* ≥ 0.14, indicating good overall fit of all the models.

**Table 4:** Associations of overweight/obesity trajectories with low self-esteem in Primary 6 (*N* = 48,558)

Trajectories	Total	General	Social	Academic/school-related	Parent/home-related
Model Ia					
Never	Reference	Reference	Reference	Reference	Reference
Late-onset	1.40 (1.19, 1.65)***	1.41 (1.27, 1.56)***	1.20 (1.08, 1.34)***	1.23 (1.10, 1.36)***	1.12 (0.98, 1.27)
Chronic	1.32 (1.14, 1.53)***	1.43 (1.31, 1.57)***	1.31 (1.20, 1.43)***	1.27 (1.17, 1.39)***	1.07 (0.96, 1.20)
Early-onset	0.91 (0.68, 1.22)	0.91 (0.76, 1.09)	0.82 (0.68, 0.98)*	0.95 (0.81, 1.13)	1.03 (0.85, 1.26)
Model IIb					
Chronic	Reference	Reference	Reference	Reference	Reference
Early-onset	0.69 (0.50, 0.95)***	0.64 (0.53, 0.77)***	0.62 (0.51, 0.76)***	0.75 (0.62, 0.90)**	0.96 (0.77, 1.20)
Never	0.76 (0.66, 0.88)***	0.70 (0.64, 0.76)***	0.76 (0.70, 0.84)***	0.79 (0.72, 0.86)***	0.93 (0.83, 1.04)
Late-onset	1.06 (0.87, 1.30)	0.98 (0.87, 1.12)	0.92 (0.81, 1.05)	0.96 (0.85, 1.09)	1.04 (0.89, 1.22)

Results are presented as adjusted odds ratios (95% confidence interval) from binomial logistic regressions, adjusted by sex, cohort, highest parental education and occupation. a Model I takes the never overweight/obesity trajectory as the reference group. b Model II takes the chronic overweight/obesity trajectory as the reference group. *P < 0.05; **P < 0.01; ***P < 0.001.

After excluding 7,716 students who were underweight in P6, 40,842 students were included in the sensitivity analysis. Based on these children, the same trajectories for weight status were identified (Supplementary Figure 1). Associations of socioeconomic characteristics with the weight status trajectories remained the same (Supplementary Table 3), and the associations of trajectories on low self-esteem were essentially the same (Supplementary Table 4). There were 35,403 children who were exactly 6-year-old in P1 and 11-year-old in P6, based on these children, the results were also essentially the same (not shown).

## Discussion

To our knowledge, this is the largest population-based longitudinal study showing that the trajectories of weight status predicted different domains of self-esteem in early adolescence. Children with unfavorable weight status trajectories were found to have higher risks of low self-esteem than others.

We showed that late-onset or chronic overweight/obesity from 6 to 11 years of age predicted low self-esteem at 11 years. Overweight/obese children, despite whether they had been underweight/normal-weighted at earlier ages or not, had similar risks of low self-esteem in the general and social domains. After commencing primary school, children begin to have extensive contact with the social environment, they become less egocentric and develop self-concept based on the interaction with teachers, peers as well as families [16]. Children in the late-onset or chronic overweight/obesity group tend to encounter more social pressure and adverse events outside their home, such as peer aggression, social marginalization, teasing and bullying [29–31]. Hence, they may be more prone to the development of poor self-esteem in their own worth and their relationship with peers.

Although there has been a controversy about whether obesity is linked to children’s actual academic performance [32, 33], our results demonstrated that children in unfavorable trajectories felt inferior in their ability to achieve academic success. They might be worried about their appearance or peer relationship and be absent-minded in class, and their negative mood may impair their enthusiasms in attending school. Meanwhile, children who had successful weight loss in primary schools, shown as being in the early-onset overweight/obesity trajectory, had a reduced risk of low self-esteem than those in the late-onset or chronic overweight/obesity trajectory. Similar results have been reported in the UK Millennium Cohort Study [12]. Further evidence is essential to explore the mechanisms of how weight changes in early childhood are associated with later self-esteem changes.

Our study also showed the existence of socioeconomic inequality in weight status trajectories. Specifically, children having lower-educated parents tended to be in the chronic or early-onset overweight/obesity group during 6-11 years. This inverse association has also been reported in American, Australian and French children [34–36]. According to Sobal’s theoretical framework, obesity is related to education primarily via knowledge and beliefs [37]. Higher-educated parents often are more health-conscious, and they directly exert positive influences on children’s dietary habits, lifestyles and daily routines via intergenerational transmission [38, 39]. Compared to children with parents working in managerial/professional positions, only those with unemployed parents had a lower risk of chronic overweight/obesity. Unemployed parents may have difficulty in affording enough food, so their children may have a higher risk of being underweight instead of overweight/obese. Given above, in developed societies like Hong Kong and elsewhere, different weight-control strategies are needed for children from families with different socioeconomic status.

Population-based longitudinal data allowed us to explore the trajectories of dynamic changes in childhood overweight/obesity. Previous studies modelled the trajectories using continuous BMI or BMI z-score [40–42]. We directly used categorical weight status and the results should be more useful and practicable. In addition to the commonly-identified trajectories using BMI or BMI z-score, including the normal-weighted, chronic obesity and late-onset obesity trajectories [42], our study identified the early-onset overweight/obese trajectory for the first time in Asian children [43], being consistent with the identified categories of weight trajectory in children from England [42, 44], Australia [41] and the US [45]. Such consistency demonstrates the validity of our study and the results should be more generalizable to other populations.

Our sample showed higher scores of social self-esteem than the normative sample. The 11-year-old children in our sample were students in P6, the last year of primary schools, whereas the 11-year-old children in the normative sample were students in secondary schools. There might be a decrease in social self-esteem when children encountered changes in social acceptance and peer relationship during their transition from primary to secondary schools [46, 47]. More in-depth studies in Asian children are needed.

This study had several limitations. Firstly, we used data already collected by SHS, although lifestyle habits such as dietary and physical activity are important confounding factors, they were time dependent and were only measured in P4 and P6 in SHS datasets, thus were not considered in this study. However, as children’s lifestyle habits mainly depend on the health consciousness and habits of parents/caregivers, their potential influences could be accounted by adjusting for their socioeconomic status in terms of parental educational level and occupation in this study. Also, the onset of puberty could affect self-esteem and the change of weight status, but such information was not available in our data. Notably, the data quality of SHS data should be high, as both the whole health assessment process and data entry followed a standardized protocol by trained nurses or SHS staff, the data collection would have a less subjective bias from the researchers and the subjects. Secondly, the predictive associations of weight status trajectory with self-esteem, although having a clear temporal sequence than cross-sectional data, could not be regarded as causal. How self-esteem affected the trajectory was not clear and in-depth studies are needed. Thirdly, obese children might have a higher risk of low self-esteem than overweight children, but due to the low prevalence of obesity (3.33%) in P6, we were unable to distinguish them. Although underweight children may have a higher risk of low self-esteem than normal weight children [27, 28], the identified trajectories and their associates had only minor changes after excluding them. Also, 3.0% of students had 2 or 3 measures of BMI (Supplementary Table 1), which should only have a slight influence on our results. Lastly, there were inevitable missing values (2.9%) in this large-scale study, however, only small effect sizes were found for the differences between the students being included and excluded in this study (Supplementary Table 5).

## Conclusions

Late-onset or chronic overweight/obesity during middle childhood predicted low general, social and academic/school-related self-esteem. Children who successfully reduced weight may have equal levels of self-esteem or even better social self-esteem than those being always underweight/normal-weight. Overweight/obese children had a vulnerability to self-esteem in non-domestic environments. We appeal for intervention studies to explore the effects of weight loss on obese children’s self-esteem.

## Supplementary Information

**Additional file 1.**

## Data Availability

The data supporting the conclusions of this study are available from the Student Health Services, Department of Health, Hong Kong SAR, but restrictions apply to the availability of these data, which were used under agreement for the current study, and so are not publicly available. Data are however available from the authors upon reasonable request and with permission of the Student Health Services, Department of Health, Hong Kong SAR.

## List of abbreviations

P1: Primary 1
P6: Primary 6
AOR: Adjusted odds ratio
BMI: Body mass index
SHS: Student Health Service
IOTF: International Obesity Task Force
SES: Socioeconomic status
CFSEI-2: Culture-Free Self-Esteem Inventories-Second Edition
AIC: Akaike information criteria
BIC: Bayesian information criterion
CI: Confidence interval
SD: Standard deviation

## References

[1] Pyszczynski T, Greenberg J, Solomon S, Arndt J, Schimel J. Why do people need self-esteem? A theoretical and empirical review. Psychol Bull. 2004;130(3):435. 10.1037/0033-2909.130.3.435. 10.1037/0033-2909.130.3.435 15122930

[2] Mann MM, Hosman CM, Schaalma HP, De Vries NK. Self-esteem in a broad-spectrum approach for mental health promotion. Health Educ Res. 2004;19(4):357–72. 10.1093/her/cyg041. 10.1093/her/cyg041 15199011

[3] Sowislo JF, Orth U. Does low self-esteem predict depression and anxiety? A meta-analysis of longitudinal studies. Psychol Bull. 2013;139(1):213–40. 10.1037/a0028931. 10.1037/a0028931 22730921

[4] Vanhalst J, Luyckx K, Scholte RH, Engels RC, Goossens L. Low self-esteem as a risk factor for loneliness in adolescence: Perceived-but not actual-social acceptance as an underlying mechanism. J Abnorm Child Psychol. 2013;41(7):1067–81. 10.1007/s10802-013-9751-y. 10.1007/s10802-013-9751-y 23661185

[5] Wild LG, Flisher AJ, Bhana A, Lombard C. Associations among adolescent risk behaviours and self-esteem in six domains. J Child Psychol Psychiatry. 2004;45(8):1454–67. 10.1111/j.1469-7610.2004.00851.x. 10.1111/j.1469-7610.2004.00851.x 15482505

[6] McGee R, Williams S. Does low self-esteem predict health compromising behaviours among adolescents? J Adolesc. 2000;23(5):569–82. 10.1006/jado.2000.0344. 10.1006/jado.2000.0344 11073698

[7] Trzesniewski KH, Donnellan MB, Moffitt TE, Robins RW, Poulton R, Caspi A. Low self-esteem during adolescence predicts poor health, criminal behavior, and limited economic prospects during adulthood. Dev Psychol. 2006;42(2):381–90. 10.1037/0012-1649.42.2.381. 10.1037/0012-1649.42.2.381 16569175

[8] Abarca-Gómez L, Abdeen ZA, Hamid ZA, Abu-Rmeileh NM, Acosta-Cazares B, Acuin C, et al. Worldwide trends in body-mass index, underweight, overweight, and obesity from 1975 to 2016: A pooled analysis of 2416 population-based measurement studies in 128.9 million children, adolescents, and adults. Lancet. 2017;390(10113):2627–42. 10.1016/S0140-6736(17)32129-3. 10.1016/S0140-6736(17)32129-3 PMC5735219 29029897

[9] Viner RM, Haines MM, Taylor SJC, Head J, Booy R, Stansfeld S. Body mass, weight control behaviours, weight perception and emotional well being in a multiethnic sample of early adolescents. Int J Obes (Lond). 2006;30(10):1514. 10.1038/sj.ijo.0803352. 10.1038/sj.ijo.0803352 16718286

[10] Franklin J, Denyer G, Steinbeck KS, Caterson ID, Hill AJ. Obesity and risk of low self-esteem: a statewide survey of Australian children. Pediatrics. 2006;118(6):2481–7. 10.1542/peds.2006-0511. 10.1542/peds.2006-0511 17142534

[11] Wang H, Leung GM, Schooling CM. Life course body mass index and adolescent self-esteem: Evidence from Hong Kong's “Children of 1997” Birth Cohort. Obesity. 2015;23(2):429–35. https://doi.org/1002/oby.20984. 10.1002/oby.20984 25557978

[12] Kelly Y, Patalay P, Montgomery S, Sacker A. BMI development and early adolescent psychosocial well-being: UK Millennium Cohort Study. Pediatrics. 2016;138(6):e20160967. 10.1542/peds.2016-0967. 10.1542/peds.2016-0967 PMC5127062 27940679

[13] Carr D, Jaffe K. The psychological consequences of weight change trajectories: Evidence from quantitative and qualitative data. Econ Hum Biol. 2012;10(4):419–30. 10.1016/j.ehb.2012.04.007. 10.1016/j.ehb.2012.04.007 PMC3414673 22580044

[14] Chen T-A, Baranowski T, Moreno JP, O’Connor TM, Hughes SO, Baranowski J, et al. Obesity status trajectory groups among elementary school children. BMC Public Health. 2016;16(1):526. 10.1186/s12889-016-3159-x. 10.1186/s12889-016-3159-x PMC4936201 27387030

[15] Gentile B, Grabe S, Dolan-Pascoe B, Twenge JM, Wells BE, Maitino A. Gender differences in domain-specific self-esteem: A meta-analysis. Rev Gen Psychol. 2009;13(1):34–45. 10.1037/a0013689.

[16] National Research Council Panel to Review the Status of Basic Research on School-Age C. In: Collins WA, editor. Development during middle childhood: The years from six to twelve. Washington (DC): National Academies Press (US), National Academy of Sciences; 1984. 10.17226/56. 25032422

[17] Biehl MC, Park MJ, Brindis CD, Pantell RH, Irwin C (2002). The health of America’s middle childhood population. San Francisco, CA: University of California.

[18] Tin SPP, Ho DSY, Mak KH, Wan KL, Lam TH. Association between television viewing and self-esteem in children. J Dev Behav Pediatr. 2012;33(6):479–85. 10.1097/DBP.0b013e31825ab67d. 10.1097/DBP.0b013e31825ab67d 22772822

[19] Cole TJ, Bellizzi MC, Flegal KM, Dietz WH. Establishing a standard definition for child overweight and obesity worldwide: international survey. BMJ. 2000;320(7244):1240. 10.1136/bmj.320.7244.1240. 10.1136/bmj.320.7244.1240 PMC27365 10797032

[20] Battle J. Culture-free self-esteem inventories (2nd ed): Austin, TX: Pro-ed; 1992.

[21] Chan YY. The normative data and factor structure of the culture-free self-esteem inventory-form a-second edition in Hong Kong adolescents. Hong Kong, CN: The University of Hong Kong; 2002. HKU Theses Online (HKUTO): b2974025. 10.5353/th_b2974025.

[22] Jones BL, Nagin DS, Roeder K. A SAS procedure based on mixture models for estimating developmental trajectories. Sociol Method Res. 2001;29(3):374–93. 10.1177/0049124101029003005.

[23] Jones BL, Nagin DS. Advances in group-based trajectory modeling and an SAS procedure for estimating them. Sociol Method Res. 2007;35(4):542–71. 10.1177/0049124106292364.

[24] Henson JM, Reise SP, Kim KH. Detecting mixtures from structural model differences using latent variable mixture modeling: A comparison of relative model fit statistics. Struct Equ Modeling. 2007;14(2):202–26. 10.1080/10705510709336744.

[25] Jay M. generalhoslem: Goodness of fit tests for logistic regression models. 2016.

[26] Hosmer Jr DW, Lemeshow S, Sturdivant RX. Applied logistic regression: John Wiley & Sons; 2013. ISBN: 0470582472.

[27] Friedlander SL, Larkin EK, Rosen CL, Palermo TM, Redline S (2003). Decreased quality of life associated with obesity in school-aged children. Arch Pediatr Adolesc Med..

[28] van den Berg PA, Mond J, Eisenberg M, Ackard D, Neumark-Sztainer D (2010). The link between body dissatisfaction and self-esteem in adolescents: Similarities across gender, age, weight status, race/ethnicity, and socioeconomic status. J Adolesc Health..

[29] Janssen I, Craig WM, Boyce WF, Pickett W (2004). Associations between overweight and obesity with bullying behaviors in school-aged children. Pediatrics..

[30] Hayden-Wade HA, Stein RI, Ghaderi A, Saelens BE, Zabinski MF, Wilfley DE. Prevalence, characteristics, and correlates of teasing experiences among overweight children vs. non-overweight peers. Obes Res. 2005;13(8):1381–92. 10.1038/oby.2005.167. 10.1038/oby.2005.167 16129720

[31] Strauss RS, Pollack HA (2003). Social marginalization of overweight children. Arch Pediatr Adolesc Med..

[32] Barrigas C, Fragoso I (2012). Obesity, academic performance and reasoning ability in Portuguese students between 6 and 12 years old. J Biosoc Sci..

[33] Taras H, Potts-Datema W (2005). Obesity and student performance at school. J Sch Health..

[34] Jansen PW, Mensah FK, Nicholson JM, Wake M. Family and neighbourhood socioeconomic inequalities in childhood trajectories of BMI and overweight: Longitudinal study of Australian children. PLoS One. 2013;8(7):e69676. 10.1371/journal.pone.0069676. 10.1371/journal.pone.0069676 PMC3720589 23936075

[35] Balistreri KS, Van Hook J (2011). Trajectories of overweight among US school children: A focus on social and economic characteristics. Matern Child Health J..

[36] Apouey BH, Geoffard P-Y (2016). Parents’ education and child body weight in France: The trajectory of the gradient in the early years. Econ Hum Biol..

[37] Sobal J (1991). Obesity and socioeconomic status: A framework for examining relationships between physical and social variables. Med Anthropol..

[38] Lindsay AC, Sussner KM, Kim J, Gortmaker S. The role of parents in preventing childhood obesity. Future Child. 2006:169–86. 10.1353/foc.2006.0006. 10.1353/foc.2006.0006 16532663

[39] Clark HR, Goyder E, Bissell P, Blank L, Peters J (2007). How do parents' child-feeding behaviours influence child weight? Implications for childhood obesity policy. J Public Health..

[40] Nonnemaker JM, Morgan-Lopez AA, Pais JM, Finkelstein EA (2009). Youth BMI trajectories: Evidence from the NLSY97. Obesity..

[41] Magee CA, Caputi P, Iverson DC (2013). Identification of distinct body mass index trajectories in Australian children. Pediatr Obes..

[42] Ziyab AH, Karmaus W, Kurukulaaratchy RJ, Zhang H, Arshad SH (2014). Developmental trajectories of body mass index from infancy to 18 years of age: Prenatal determinants and health consequences. J Epidemiol Community Health..

[43] Haga C, Kondo N, Suzuki K, Sato M, Ando D, Yokomichi H, et al. Developmental trajectories of body mass index among Japanese children and impact of maternal factors during pregnancy. PLoS One. 2012;7(12):e51896.10.1371/journal.pone.0051896. 10.1371/journal.pone.0051896 PMC3521723 23272187

[44] Mustillo S, Worthman C, Erkanli A, Keeler G, Angold A, Costello EJ. Obesity and psychiatric disorder: Developmental trajectories. Pediatrics. 2003;111(4):851–9.10.1542/peds.111.4.851. 10.1542/peds.111.4.851 12671123

[45] Huang DY, Lanza HI, Wright-Volel K, Anglin MD (2013). Developmental trajectories of childhood obesity and risk behaviors in adolescence. J Adolesc..

[46] Coelho VA, Romão AM. The impact of secondary school transition on self-concept and self-esteem. Rev de Psicodidactica (English ed). 2017;22(2):85–92. 10.1016/j.psicoe.2016.10.001.

[47] Poorthuis AMG, Thomaes S, van Aken MAG, Denissen JJA, Orobio de Castro B. Dashed hopes, dashed selves? A sociometer perspective on self-esteem change across the transition to secondary school. Soc Dev. 2014;23(4):770–83. 10.1111/sode.12075.

