# Supplementary material for: Late-onset or chronic overweight/obesity predicts low self-esteem in early adolescence: a longitudinal cohort study
Source: BMC Public Health. 2022 Jan 6;22:31. doi: 10.1186/s12889-021-12381-5 (PMC8740381; doi:10.1186/s12889-021-12381-5)
Supplement: Supplementary file 1 — Additional file 1. [file 12889_2021_12381_MOESM1_ESM.docx]

Supplementary Table 1 Description of students having repeated measures of body mass index

| Cohort | Number of repeated measures of body mass index, *n* (%) | | | | | |
| --- | --- | --- | --- | --- | --- | --- |
|  | 2 | 3 | 4 | 5 | 6 | Total |
| 1995/96 | 208 (0.9) | 505 (2.1) | 1145 (4.7) | 3288 (13.5) | 19,229 (78.9) | 24,375 |
| 1996/97 | 211 (0.9) | 492 (2.0) | 1038 (4.3) | 3108 (12.9) | 19,334 (80.0) | 24,183 |
| Total | 419 (0.9) | 997 (2.1) | 2183 (4.5) | 6396 (13.2) | 38,563 (79.4) | 48,558 |

Supplementary Table 2 Model fit indices used to identify the optimal number of latent classes (*N* = 48,558)

| Number of latent classes (groups) | AIC | BIC | ssBIC | Entropy | LMR (*P*) |
| --- | --- | --- | --- | --- | --- |
| 1 | -127,148.20 | -127,145.60 | -127,132.40 | - |  |
| 2 | -71,002.57 | -70,994.76 | -70,955.18 | 0.961 | <0.001 |
| 3 | -67,851.84 | -67,838.82 | -67,772.86 | 0.930 | <0.001 |
| 4 | -65,729.04 | -65,705.58 | -65,586.91 | 0.935 | <0.001 |
| 5 | -65,399.54 | -65,370.87 | -65,225.82 | 0.907 | 0.19 |

AIC: Akaike information criteria. BIC: Bayesian information criterion. ssBIC: Sample-size adjusted BIC. LMR (*P*): The *P* values calculated using the Lo-Mendell-Rubin likelihood ratio test.

Supplementary Figure 1 Developmental trajectories of overweight/obesity in Hong Kong children (without underweight children) (*N* = 40,842)

Supplementary Table 3 Associations of baseline characteristics with overweight/ obesity trajectories (without underweight children) (*N* = 40,842)

| Characteristics | Late-onset | Chronic | Early-onset |
| --- | --- | --- | --- |
| Sex |  |  |  |
| Female | Reference | Reference | Reference |
| Male | 2.30 (2.14, 2.47)*** | 1.72 (1.62, 1.83)*** | 0.85 (0.77, 0.94) |
| Parental educational level |  |  |  |
| Tertiary | Reference | Reference | Reference |
| Secondary | 1.16 (1.01, 1.34)* | 1.16 (1.03, 1.30)** | 0.80 (0.67, 0.96)* |
| Primary/below | 1.20 (1.02, 1.41)* | 1.18 (1.02, 1.35)* | 0.71 (0.57, 0.89)*** |
| Parental occupation |  |  |  |
| Managerial/professional | Reference | Reference | Reference |
| Clerical/service industry | 0.99 (0.90, 1.10) | 0.99 (0.91, 1.08) | 1.01 (0.87, 1.18) |
| Manual job | 0.97 (0.87, 1.07) | 0.97 (0.89, 1.06) | 1.05 (0.91, 1.22) |
| Unemployed | 1.21 (0.97, 1.50) | 0.78 (0.62, 0.97)** | 0.82 (0.57, 1.20) |
| Cohort |  |  |  |
| 1995/96 | Reference | Reference | Reference |
| 1996/97 | 0.97 (0.91, 1.04) | 1.05 (0.99, 1.12)* | 1.18 (1.07, 1.30)** |

Results were presented as adjusted odds ratios (95% confidence interval) from multinomial logistic regression, with the never overweight/obesity trajectory taken as the reference category. **P* < 0.05; ***P* < 0.01; ****P* < 0.001.

Supplementary Table 4 Associations of overweight/obesity trajectories with low self-esteem in Primary 6 (without underweight children) (*N* = 40,842)

| Trajectories | Total | General | Social | Academic/school-related | Parent/home-related |
| --- | --- | --- | --- | --- | --- |
| Model Ia |  |  |  |  |  |
| Normal | Reference | Reference | Reference | Reference | Reference |
| Late-onset | 1.38 (1.17, 1.63)*** | 1.42 (1.28, 1.58)*** | 1.26 (1.13, 1.41)*** | 1.21 (1.09, 1.35)*** | 1.09 (0.96, 1.24) |
| Chronic | 1.31 (1.13, 1.52)*** | 1.45 (1.32, 1.59)*** | 1.38 (1.26, 1.52)*** | 1.26 (1.15, 1.38)*** | 1.05 (0.94, 1.18) |
| Early-onset | 0.90 (0.67, 1.21) | 0.92 (0.77, 1.10) | 0.86 (0.72, 1.04) | 0.94 (0.79, 1.11) | 1.01 (0.82, 1.23) |
| Model Ib |  |  |  |  |  |
| Chronic | Reference | Reference | Reference | Reference | Reference |
| Early-onset | 0.69 (0.50, 0.94)*** | 0.64 (0.52, 0.77)*** | 0.62 (0.51, 0.76)*** | 0.74 (0.62, 0.89)** | 0.96 (0.77, 1.19) |
| Never | 0.76 (0.66, 0.89)*** | 0.69 (0.63, 0.76)*** | 0.72 (0.66, 0.79)*** | 0.79 (0.73, 0.87)*** | 0.95 (0.85, 1.07) |
| Late-onset | 1.05 (0.86, 1.29) | 0.98 (0.87, 1.11) | 0.91 (0.80, 1.04) | 0.96 (0.85, 1.09) | 1.04 (0.88, 1.21) |

Results were presented as adjusted odds ratios (95% confidence interval) from binomial logistic regressions, adjusted by sex, cohort, highest parental education and occupation. a. Model I takes the never overweight/obesity trajectory as the reference group. b. Model II takes the chronic overweight/obesity trajectory as the reference group. **P* < 0.05; ***P* < 0.01; ****P* < 0.001.

Supplementary Table 5 Comparisons of characteristics of students being included and excluded in this study, *n* (%)

| Characteristics | Included  (*n* = 48,558) | Excluded  (*n* = 1,447) | *P* | Effect size ^a^ |
| --- | --- | --- | --- | --- |
| Age, Mean±SD (years) |  |  |  |  |
| Sex |  |  | <0.001 | 0.02 |
| Female | 24297 (50) | 632 (43.7) |  |  |
| Male | 24261 (50) | 815 (56.3) |  |  |
| Parental educational level (missing=150) | |  | 0.31 | 0.007 |
| Tertiary | 4417 (9.1) | 119 (9.2) |  |  |
| Secondary | 35711 (73.5) | 974 (75.1) |  |  |
| Primary/below | 8430 (17.4) | 204 (15.7) |  |  |
| Parental occupation (missing=57) | |  | 0.31 | 0.009 |
| Managerial/professional | 10182 (21) | 276 (19.9) |  |  |
| Clerical/service industry | 15252 (31.4) | 468 (33.7) |  |  |
| Manual job | 21918 (45.1) | 615 (44.2) |  |  |
| Unemployed | 1206 (2.5) | 31 (2.2) |  |  |
| Cohort |  |  | 0.16 | 0.006 |
| 1995/96 | 24375 (50.2) | 699 (48.3) |  |  |
| 1996/97 | 24183 (49.8) | 748 (51.7) |  |  |
| Weight status (missing=10) |  |  | 0.83 | 0.004 |
| Underweight | 7716 (15.9) | 219 (15.2) |  |  |
| Normal | 31336 (64.5) | 934 (64.7) |  |  |
| Overweight | 7884 (16.2) | 239 (16.6) |  |  |
| Obesity | 1615 (3.3) | 52 (3.6) |  |  |

SD: Standard deviation. Missing data were excluded.

^a^ Cohen’s d for continuous age: small=0.10, medium=0.30, and large=0.50. Cohen’s w for categorical variables: small=0.10, medium=0.30, and large=0.50.
